# Changes in alcohol use and relationship satisfaction in Norwegian couples during pregnancy

**DOI:** 10.1186/1747-597X-8-5

**Published:** 2013-01-28

**Authors:** Sonja Mellingen, Torbjørn Torsheim, Frode Thuen

**Affiliations:** 1 Centre for Evidence-Based Practice, Bergen University College, Møllendalsveien 6, 5009, Bergen, Norway; 2 Department of Psychosocial Science, Faculty of Psychology, University of Bergen, PO Box 7800, 5020, Bergen, Norway

**Keywords:** Alcohol consumption, Pregnancy, Relationship satisfaction, Mothers, Change in consumption levels, First-time fathers, Social roles, Gender equality, Drinking patterns, Heavy episodic drinking (HED)

## Abstract

**Background:**

Numerous studies have documented a profound reduction in alcohol use among pregnant women, whereas research on expectant fathers has been scarce. The aim of this study was to measure changes in alcohol consumption from before pregnancy to 17 weeks in gestation for mothers and fathers, differentiating between parents with and without any previous children, and to measure how level and change in alcohol consumption into early pregnancy was associated with relationship satisfaction.

**Methods:**

The data collection was conducted as part of the Norwegian Mother and Child Cohort Study (MoBa) at the Norwegian Institute of Public Health. This cohort now includes 108 000 children, 90 700 mothers and 71 500 fathers recruited from 1999 to 2008. The present study comprises 82 362 couples. Alcohol consumption was assessed using a questionnaire including items about usual drinking frequency, quantities, and number of occasions with heavy episodic drinking (HED). Relationship satisfaction was measured by five items scored on a Likert agreement scale.

**Results:**

The findings indicate that both mothers and fathers reduce their drinking significantly during pregnancy. Reduction was apparent for all three measures of alcohol consumption. First-time fathers reduced their alcohol consumption more than experienced fathers, from initially higher levels. The gap between the fathers and their pregnant partner was greater for first-time parents compared to parents with previous children. Drinking pre-pregnancy and relationship satisfaction during pregnancy were weakly related within each partner, whereas no association across partners was observed.

**Conclusions:**

Both expectant mothers and fathers changed their alcohol consumption patterns when expecting a child. Almost all mothers stopped drinking, whereas fathers reduced their drinking to a considerable degree. Relationship satisfaction was only slightly related to their drinking patterns. The findings may have important policy implications, mainly with regard to developing alcohol preventive strategies.

## Background

Alcohol consumption changes across life phases [1] and cohorts [2,3]. Young groups are the heaviest drinkers, and the highest drinking level usually occurs early in the twenties [2,4]. Towards the end of the twenties there is a considerable drop [4-6]. This corresponds with two major life events often taking place during this particular phase; marriage and transition to parenthood. When a couple get married they usually begin to decrease their consumption, [7,8], a phenomenon referred to as “marriage effect” [9]. During pregnancy it is usually not the physical changes involved in becoming pregnant (aside from probably morning sickness) that produces the observed reduction in alcohol consumption [10-13], but more likely the women’s own decision to reduce their drinking for the health of the baby*.* In keeping with this, Alvik et al. [10] found that 85% of women reduced their alcohol consumption at pregnancy recognition, with fetal welfare being the main reason.

Generally, research focusing on expectant fathers has been scarce [14]. Thus, little is known about expectant fathers’ adaptation to their partner’s pregnancy, including the extent to which they reduce their own alcohol use when expecting a child. The few studies that have measured any changes in fathers’ drinking pattern indicate that the majority do not reduce their alcohol use when their partner becomes pregnant [3,4,15,16]. These studies are based on quite small and highly selected samples [3,15] or on data collected in the 1970s to the middle of the 1990s [4,16], where the health risks related to alcohol in pregnancy were less acknowledged and emphasized in public. Therefore, the findings cannot be generalized to other populations of expectant fathers. In keeping with this, fathers’ role during pregnancy might differ with different levels of gender equality. Thus, a high degree of gender equality might be reflected in a tendency for men to adapt to pregnancy in similar ways to women. Particularly of relevance are men’s perceptions of what is expected of them in their role as expectant father, taking part in the preparation for birth and taking care of their pregnant partner.

To the extent that men experience such expectations and adapt to them, this may indicate that they develop a new social role. According to classical role theory, many social roles are associated with a more structured life and consequently fewer opportunities to engage in heavy drinking [17]. Thus, one might expect that men in a culture with a relatively high level of gender equality, such as in Norway [18], would decrease their alcohol use during their partner’s pregnancy. To our knowledge, there are no publications of any such effects of gender equality. However, findings from a study across several states in the USA suggest that higher levels of gender equality are associated with lower overall alcohol consumption for both men and women [19]. The social pressure for expectant fathers to abstain from drinking is obviously not as strong as for pregnant women. Therefore, one should not expect men to decrease their alcohol use to the same extent as their female partners.

Fathers’ drinking pattern is important for at least two reasons. First, it may influence the pregnant mother’s alcohol use, since heavy drinking among fathers has been related to high-risk drinking among expectant mothers [15,19]. In general populations, male partners have been found to affect their female partners’ alcohol use [20,21], Second, changes in the drinking pattern of either of the partners may affect their relationship. This is based on the general notion that alcohol use and the quality of the relationship are related [22]. Such associations have been reported in various samples; clinical groups [23], couples who have just separated [24], and in population-based samples [25]. One particular aspect of interest has been any discrepancy in drinking pattern between the partners, since such discrepancy seems to be associated with poor relationship satisfaction [26-28]. Discrepancy in drinking patterns may be of special relevance during pregnancy, since most pregnant women abstain from alcohol use, or reduce their consumption considerably, whereas most men continue their previous drinking habits.

Based on our selective review of the literature, some gaps in knowledge can be identified. First, even if expectant fathers in cultures with a relatively high level of gender equality may reduce their alcohol use, they are not likely to reduce it as much as pregnant women. Thus pregnancy is still expected to lead to an increase in the difference in alcohol use between men and women. Previous studies in general populations have found male partners to affect their female partners’ alcohol use [20,21], whereas this might be otherwise among expectant parents, due to the strong pressure on women to abstain from alcohol during pregnancy. To our knowledge there are no publications explicitly comparing changes in men’s and women’s alcohol use during pregnancy, or assessing the extent to which their changes in alcohol use during this phase influence the couple’s relationship satisfaction. Second, with a few exceptions [29], there has been little focus on the distinction between first-time parents and experienced parents, thus leaving it open whether any changes in alcohol use can be related to the mere condition of being pregnant or to being in the transition to parenthood. More knowledge about these issues may have policy implications, particularly for preventive strategies. If expectant fathers actually reduce their alcohol use in parallel with their pregnant partner, this could influence total alcohol consumption in the population, and have a non-specific positive public health effect. Moreover it may reduce the potential risk of harm to others caused by alcohol consumption. Consequently health education measures might also include the fathers’ drinking habits as a target.

The present paper is based on a national population study among Norwegian parents. Norway is generally considered to be a “dry country” with regard to alcohol consumption culture, indicating a risky drinking style of high quantities [30] on fewer occasions [31], although a transition towards a more “wet” drinking culture more typical for the southern parts of Europe has emerged during the last few decades [32]. The first aim of the study was to assess patterns of alcohol use of pregnant women and their partners that characterize a “dry” versus a “wet” drinking culture: frequency of drinking occasions, typical number of units consumed per occasion, and number of times five or more units were consumed on one occasion. The second aim was to assess the strength of the relationship between parental status (first-time parents versus experienced parents) and alcohol use. The final aim was to assess the extent to which the partners’ individual alcohol use before and during pregnancy was associated with their relationship satisfaction.

## Method

### Procedures and design

The data collection was conducted as part of the Norwegian Mother and Child Cohort Study (MoBa) [33]. MoBa is a prospective population-based pregnancy cohort study conducted by the Norwegian Institute of Public Health. Participants were recruited to the study from all over Norway through a postal invitation in connection with a routine ultrasound examination offered to all pregnant women in Norway at 17–18 weeks of gestation (http://www.fhi.no/morogbarn) from 1999–2008, and 38.5% of invited women consented to participate. The cohort now includes 108 000 children, 90 700 mothers and 71 500 fathers. All pregnant women in Norway were invited to participate, provided that they could read Norwegian. Prevalence estimates of exposure and outcomes have been tested. No estimates were biased due to self-selection in these previous analyses [34].

The current study is based on version 6 of the quality-assured data files released for research, including 101 111 pregnancies. Informed consent was obtained from each MoBa participant on recruitment. The study was approved by the Regional Committee for Medical Research Ethics and the Norwegian Data Inspectorate.

The data were collected by means of self-administered questionnaires, received by mail at home, and delivered at eight time waves from approximately 17 weeks of gestation (t1) to when the child was seven years old (t8). This included three waves during pregnancy and five after the child was born. In the present study we used data from the first wave (t1,17 weeks of gestation). Based on the information the mothers gave about their partners, fathers were recruited and received one self-administered questionnaire by mail at home. This was at approximately 17–18 weeks of gestation.

### Participants

The present results include couples participating in the MoBa study for the first-time (82 362 pregnancies, 82 362 mothers and 62 281 fathers). Mothers living alone were excluded, and multi-time participating mothers were included with only their first participating pregnancy. Analyses were based on information about both partners, however in some cases relying on information from the mother only (e.g. length of relationship, marital status). This accounts for high missing values for some of the demographic variables. Questions about individual behaviour or experiences, such as alcohol consumption and relationship satisfaction, relied on individual answers from each partner. Table 1 shows participants’ characteristics. Based on mothers’ reports, 52.2% of couples were cohabiting, and 47.8% were married, with a mean duration for the relationship of 6.3 years. A total of 54.4% of the couples were first-time parents, and 45.6% were experienced parents. Compared with official Norwegian statistics for women and men aged between 16–49 years for 2008, the level of education was higher for the sample than for the general population (official statistics in brackets): mothers with compulsory school, 7.8% (28.1%), vocational school, 28.5% (35.7%), three year college, 40.8% (29.8%) and university higher education, 22.9% (6.4%). Fathers with compulsory school, 10.4% (32.1%), vocational school, 38.9% (42.6%), three year college, 26.8% (17.9%) and university higher education, 23.9% (7.4%).

**Table 1:** Descriptive for study variables

**Variable**	**Mean/percentage**	**95% CI**	**n**	**Missing**
**Marital status**				
Cohabiting	52.2%	(51.9 to 52.6)		
Married	47.8%	(47.4 to 48.1)		
**Parenting status**			78918	3444
Experienced parents	45.6%	(45.2 to 45.9)		
First-time parents	54.4%	(54.1 to 54.8)		
**Ethnicity**			78400	3962
Norwegian	88.5%	(88.2 to 88.7)		
Non-Norwegian	11.5%	(11.3 to 11.8)		
**Ethnicity of parents**			78292	4070
Norwegian	83.8%	(83.5 to 84.0)		
Non-Norwegian	16.2%	(16.0 to 16.5)		
**Education mother**			78076	4286
Compulsory school	7.8%	(7.6 to 8.0)		
Vocational school	28.5%	(28.2 to 28.8)		
Three-year College	40.8%	(40.5 to 41.2)		
University higher education	22.9%	(22.6 to 23.2)		
**Education father**			59784	2497^a^
Compulsory school	10.4%	(10.2 to 10.7)		
Vocational school	38.9%	(38.5 to 39.3)		
Three-year College	26.8%	(26.5 to 27.2)		
University higher education	23.9%	(23.5 to 24.2)		

Note. ^a^ The total number of participating fathers was 62281.

### Measures and variables

#### Alcohol consumption

Alcohol consumption, before and during pregnancy was measured according to monthly frequency, monthly heavy episodic drinking (HED) and number of alcohol units (AU) per drinking occasion. The reference period for mothers was three months before pregnancy and for fathers six months before pregnancy. The alcohol consumption measures were: *How often did you consume alcohol in the three months/six months before you became pregnant/before your partner became pregnant*, and *How often do you consume alcohol now during the pregnancy?* The response categories were; *1) approximately 6–7 times a week, 2) approximately 4–5 times a week, 3) approximately 2–3 times a week, 4) approximately once a week, 5) approximately 1–3 times a month, 6) less than once a month* and *7) never*. Number of HED episodes was defined as 5 or more alcohol units on a single occasion; *How often if ever, on a monthly basis, have you experienced drinking five alcohol units or more, during the last three months/six months, before pregnancy, and in pregnancy?* The response categories were; *1) several times a week; 2) once a week; 3) 1–3 times per month; 4) less than once a month* and *5) never.*

Amount of drinking, measured in alcohol units, where 1 unit was equivalent to 1.5 cl pure alcohol (1 bottle of alcopop/cider, 1 glass of beer(1/3 litre), 1 wine glass of red wine or white wine, 1 sherry glass of sherry or other fortified wine, 1 glass with a single measure of spirit or liquor); *How many alcohol units did you normally drink when you consume alcohol in the three months/six months before you/your partner became pregnant* and *How many alcohol units do you drink now that you are/your partner is pregnant?* The response categories were; *10 or more, 7–9, 5–6, 3–4, 1–2* and *less than 1*. In the analysis, all item values were re-coded to reflect the continuous underlying metric (1–2 episodes coded as 1.5, 3–4 episodes coded as 3.5, 5–6 episodes coded as 5.5, 7–9 episodes coded as 8 and 10 or more episodes coded as 10).

#### Partner relationship

Relationship satisfaction (RS) was measured using five items scored on a Likert agreement scale ranging from 1 “totally agree” to 6 “don’t agree at all”. The scale originally contained 10 items, showing good psychometric properties (Cronbach’s Alpha = 0.91) [35], and was highly correlated with the Quality of Marriage Index (*r* = 0.92) [36]. Only a subset of the sample received the full ten item scale. The five-item version was available for all the participants in the MoBa study, and was used for the present study. The five item version included the following items: *“My partner and I have problems in our relationship”; “I am very happy in my relationship”; “My partner is generally very understanding”; “I am satisfied with my relationship with my partner”* and *“We agree about child rearing issues”*, with a Cronbach’s alpha of 0.85 for mothers and 0.82 for fathers. The five item scale was highly correlated with the ten item scale (mother: *r* = 0.98; father: *r* = 0.96 ). The duration of relationships was measured using the women’s report of how long they had had a sexual relationship with their current partner before the pregnancy.

#### Statistical procedure

All statistical procedures were conducted in Mplus 6.11 [37]. The key population characteristic to model was the mean level and variance of alcohol consumption pre-pregnancy, and the mean level and variance of change in alcohol consumption associated with entry into pregnancy. Two data points preclude analysis of the shape of change, but allow formulation of linear change and a variation across the mean slope and intercept (for a didactic example see, 37). This model allows for regressions of pre-pregnancy levels (intercept) and change (slope) on independent couple characteristics. To isolate the effects of transition into parenthood the initial analysis was run as a two-group structural equation model. The first group consisted of “first-time parents” and the second group consisted of “experienced parents” with previous children. By including parent status as a group variable, the group difference in pre-pregnancy levels and change into pregnancy between first-time parents and experienced parents would represent the unadjusted transition effect on pre-pregnancy levels and change in alcohol consumption. Adjustments for third variables were done by including other assumed confounders such as marital status, duration of relationship, age, level of education and ethnicity.

Since reports from both parents were available, the pre-pregnancy levels and change could be modelled within an actor-partner-interdependence (APIM) framework, with simultaneous modelling of both partners’ pre-pregnancy levels and change in alcohol use [38]. One of the main goals of APIM is to account for the interdependence of dyadic data. This is particularly relevant in studying the reciprocal relationships, and whether mothers’ change in alcohol use also affects the father.

The final set of analysis modeled the impact of alcohol consumption on couples’ relationship satisfaction. In this model mothers’ and fathers’ relationship satisfaction was specified as two correlated latent factors, measured each by five items. To account for the residual dependency of fathers’ and mothers’ relationship satisfaction a multivariate approach was followed, allowing the residuals of the two latent factors to be correlated. The two latent variables were regressed on background characteristics as well as mothers’ and fathers’ alcohol pre-pregnancy level and change in alcohol use, with simultaneous modeling of the independent effects of HED, frequency of drinking and typical amount of drinking per drinking occasion. Abstaining couples were not included in the analysis, thus the available sample for analysis was 60 075 couples.

Due to revisions of the questionnaires between cycles of the MOBA study, complete information on alcohol outcomes was not collected for all fathers. For 34900 fathers, only partial information on the current alcohol outcomes was collected. For fathers with partially collected information, parameter estimation was based on all available data, with missing data estimated using full information maximum likelihood, assuming a Missing at Random (MAR) missing mechanism.

This assumption implies that the missing data mechanism, conditional on observed variables, is unrelated to unobserved variables. Conditional on the observed data, there are no unobserved variables that account for missingness. Sensitivity analyses indicated very minor differences between analyses conducted on the full information sample and the complete case sample.

The number of missing values differed across analysis, depending on the number of observed covariates. Since the current models specified relationship satisfaction and pre-pregnancy levels and change in alcohol use as latent variables, cases with available information on any of these observed alcohol variables were retained in the analysis. However, cases with missing information on any exogenous observed indicators (x-variables) such as gender, education, ethnicity, age, and relationship duration were excluded from the analysis. Analyses including observed covariates were thus based on a smaller effective sample (*n* = 52 275) than analyses without such covariates (*n =* 60 068). Seven cases had missing values for all variables and were not included in the analyses. To accommodate deviations form normality in the observed dependent variables, models for continuous independent variables were estimated with the Yuan-Bentler robust maximum likelihood estimator [39]. The analysis of missing father data was based on robust weighted least square (WLSMV) using a probit link function.

## Results

### Missing data

Based on mother reports of marital status, the pregnancies of 82 362 couples were available for analyses. Of these 82 362 couples 62 281 fathers participated with data. To test whether couples with non-responding fathers differed from couples with data from both partners, a probit regression model was fitted using father participation (coded 1) as the dependent variable. The results from this analysis are shown in Table 2. The table shows standardized regression coefficients with Wald tests on 82348 error degrees of freedom, and the associated p-value for the Wald statistic. Father participation was higher for first-time parent couples and higher for couples where mothers were more satisfied with the relationship, and also for mothers with higher education. Furthermore, ethnic non-Norwegians, higher number of alcohol units, and higher alcohol frequency was associated with lower father participation.

**Table 2:** Father participation regressed on mother characteristics

	**B**	**t**^**†**^	**p**
Age mother	0.02	3.472	0.001
Relationship duration	−0.051	−12.136	0.001
First-time parents	0.143	26.087	0.001
Married	0.03	5.849	0.001
Education	0.067	12.494	0.001
Relationship satisfaction	0.081	16.638	0.001
Ethnicity	−0.029	−5.563	0.001
HED mother before pregnancy	0.005	0.792	0.428
HED mother in pregnancy	0.000	−0.051	0.960
Unit alcohol per occasion before pregnancy	−0.004	−0.574	0.566
Unit alcohol per occasion in pregnancy	−0.020	−3.351	0.001
Frequency of alcohol before pregnancy	0.020	3.102	0.002
Frequency of alcohol in pregnancy	−0.036	−11.84	0.001

Note. ^†^ Wald test on *t*(82349).

### Pre-pregnancy level and change in alcohol use

**Figure 1:**
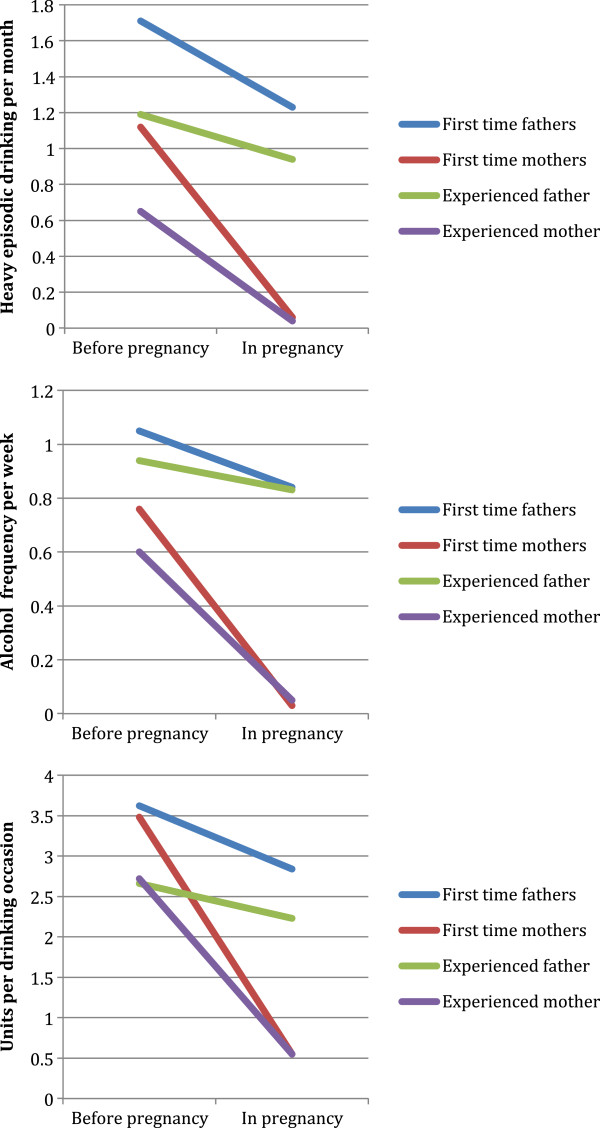
Alcohol use pre-pregnancy and in pregnancy.

To assess patterns of alcohol use of pregnant women and their partners we estimated pre-pregnancy levels and change in alcohol use among first-time and experienced parents. Figure 1 shows the estimated levels of alcohol use before pregnancy, and the estimated change into pregnancy, for heavy episodic drinking (HED), frequency of alcohol use, and number of alcohol units per occasion. It can be seen that first-time mothers had a sharp decline in their alcohol use, with amount of change largely corresponding to their pre-pregnancy levels of alcohol use. Also their male partners changed from before pregnancy into the pregnancy. First time fathers cut their heavy episodic drinking with 0.48 times per month, their frequency of alcohol consumption 0.21 times per week, and their number alcohol units per drinking occasion with 0.78 units (see Table 3). Analysis of complete abstinence revealed that among drinking first-time parents 90.0% of the mothers and 2.2% of the fathers completely stopped drinking alcohol at this point in pregnancy. Corresponding findings among experienced parents were 82.5% and 2.1% for mothers and fathers, respectively.

**Table 3:** Mean HED, frequency, AU pre-pregnancy and change into pregnancy by first-time and experienced parents

	**First-time parents (n = 34966)**				**Experienced parents (n = 25102)**			
	**Pre-pregnancy**	**95% CI**	**Change**	**95% CI**	**Pre-pregnancy**	**95% CI**	**Change**	**95% CI**
HED father	1.71	(1.69 to 1.73)	−0.48	(−0.49 to −0.46)	1.19^†^	(1.16 to 1.21)	−0.25^***^	(−0.26 to −0.23)
HED mother	1.12	(1.11 to 1.14)	−1.06	(−1.07 to −1.04)	0.65^†^	(0.64 to 0.66)	−0.61^***^	(−0.62 to −0.60)
Frequency father	1.05	(1.04 to 1.06)	−0.21	(−0.22 to −0.21)	0.94^†^	(0.92 to 0.95)	−0.11^***^	(−0.12 to −0.11)
Frequency mother	0.76	(0.75 to 0.77)	−0.73	(−0.74 to −0.72)	0.60^†^	(0.59 to 0.61)	−0.55^***^	(−0.56 to −0.54)
Units father	3.62	(3.59 to 3.66)	−0.78	(−0.80 to −0.75)	2.66^†^	(2.62 to 2.71)	−0.43^***^	(−0.45 to −0.40)
Units mother	3.48	(3.45 to 3.50)	−2.93	(−2.95 to −2.91)	2.72^†^	(2.70 to 2.75)	−2.17^***^	(−2.19 to −2.14)

Note. ^†^ Wald test of differences between first time parents and experienced parents pre-pregnancy level of alcohol outcomes on *t*(60056) is statistically significant at the .001 level of significance. *** Wald test of differences between first time parents and experienced parents change in alcohol outcomes on *t*(60056) is statistically significant at the .001 level of significance.

In line with the second aim of the study, we tested the difference in alcohol use between first-time parents and experienced parents. Moderation tests revealed significant differences between first-time parents and experienced parents on all alcohol outcomes. First-time parents’ pre-pregnancy alcohol use was clearly higher than in the group of experienced parents (differences pre-pregnancy, first-time fathers – experienced fathers: HED 0.52 *t(60056)* = 30.31, p < .001 ; frequency 0.11, *t(60056)* = 13.86, p < .001; units per occasion 0.96, *t(60056)* = 31.12, p < .001). Due to their initial higher level, first-time fathers had a stronger reduction in alcohol use into pregnancy, as compared to experienced fathers (difference change, first-time fathers – experienced fathers: HED 0.23, *t(60056)* = 22.06, p < .001; frequency 0.10, *t(60056)* = 23.57, p < .001; units per occasion 0.35, *t(60056)* = 20.22, p < .001).

Subsequent analyses not reported in tables revealed that pre-pregnancy levels and change in alcohol use, varied as a function of couple characteristics, however, the associations were generally weak. To summarize; married couples had lower pre-pregnancy levels of alcohol use, and showed a less reduction into pregnancy, as compared to cohabiting couples. Also ethnicity showed a differential association with alcohol use: Non-Norwegians had lower number of HED per month, and a lower number of alcohol units per drinking episode, but a higher frequency of drinking as compared to people with Norwegian as their mother tongue. In general higher education was associated with higher pre-pregnancy drinking frequency, but lower number of units per drinking occasions. Adjustment for these third-variables did not have any substantial impact on the differences between first-time and experienced parents’ pre-pregnancy levels and change in alcohol use.

Table 4 shows the Pearson-r correlation between the fathers’ and mothers’ pre-pregnancy levels and change in alcohol use into pregnancy, for each of the indicators of HED, frequency of drinking and units of alcohol per drinking episode. All correlations were statistically significant at the 5% level of significance. It can be seen that the alcohol use in pre-pregnancy for first-time mothers and fathers was moderately correlated, ranging from *r(34964)* = 0.41 for units per drinking episode to *r(34964)* = 0.52 for drinking frequency. Another noteworthy result was the high inverse correlation between pre- pregnancy levels of alcohol use and change in alcohol use for mothers. The correlation indicates that mothers’ change of alcohol use was clearly dependent on their initial levels. A similar pattern was observed for the fathers, although the correlation between pre-pregnancy levels and change was of moderate magnitude. Fathers’ change in alcohol use was weakly to moderately correlated with mothers’ change in alcohol use, with strongest association found for frequency of alcohol use (first-time fathers: *r(34964)* = 0.31; experienced fathers: *r(25100)* = 0.25).

**Table 4:** **Correlation between pre-pregnancy and change in alcohol drinking patterns within partners (first-time parents below diagonal, experienced parents above diagonal)**^**a**^

	**Pre-pregnancy**	**Change**	**Pre-pregnancy**	**Change**
		**Father**	**Father**	**Mother**	**Mother**
**HED**				
Pre-pregnancy father		−0.49	0.28	−0.28
Change father	−0.57		−0.19	0.18
Pre-pregnancy mother	0.41	−0.23		−0.97
Change mother	−0.40	0.23	−0.96	
**Frequency of drinking**				
Pre-pregnancy father		−0.41	0.47	−0.45
Change father	−0.52		−0.25	0.25
Pre-pregnancy mother	0.52	−0.32		−0.97
Change mother	−0.51	0.31	−0.99	
**Units per drinking occasion**				
Pre-pregnancy father		−0.44	0.30	−0.30
Change father	−0.46		−0.16	0.16
Pre-pregnancy mother	0.41	−0.18		−0.98
Change mother	−0.40	0.18	−0.98	

Note. ^a^ All correlations statistically significant at the .05 level of significance using Fisher Z-test for H_0_*r*(60066) = 0.

### Alcohol use and relationship satisfaction in pregnancy

In line with the final aim of the study, relationship satisfaction was regressed on pre-pregnancy levels and change in HED, frequency of alcohol use and units per drinking occasion, adjusting for relevant couple characteristics. Since mothers’ change in alcohol consumption indicators correlated highly with pre-pregnancy levels only pre-pregnancy drinking for mothers was included in the model, to avoid multicollinearity. Table 5 shows the statistically significant results of a regression model with relationship satisfaction regressed on alcohol use among first-time and experienced parents, omitting non-significant paths. The table shows unstandardized regression coefficients with Wald test on 52239 error degrees of freedom, and the associated p-value for the *t*-statistic. In general, alcohol use was inversely related to relationship satisfaction. For first-time mothers, mothers’ pre-pregnancy drinking was associated with lower relationship satisfaction. There were also partner effects, to the extent that mothers’ pre-pregnancy levels of drinking were related to fathers’ relationship satisfaction. In contrast, fathers’ alcohol use did not predict mothers’ relationship satisfaction. Change in fathers drinking was not significantly related to relationship satisfaction.

**Table 5:** Latent regression of relationship satisfaction on alcohol use in mothers and fathers

	**First-time**			**Experienced**		
	**B**	**t**^**†**^	**p**	**B**	**t**^**†**^	**p**
	DV: Mothers’ relationship satisfaction					
HED mother pre-pregnancy	−0.035	−3.867	0.001	−0.047	−2.864	0.004
Frequency mother pre-pregnancy	−0.052	−2.36	0.018	−0.072	−2.619	0.009
Units mother pre-pregnancy	−0.016	−4.063	0.001	−0.019	−3.137	0.002
	DV: Fathers’ relationship satisfaction					
HED father pre-pregnancy	−0.011	−2.124	0.034	−0.019	−2.045	0.041
Frequency father pre-pregnancy	−0.015	−2.784	0.005	−0.012	−1.803	0.071
Frequency mother pre-pregnancy	−0.040	−2.317	0.021	0.014	0.672	0.501
Units mother pre-pregnancy	−0.010	−2.926	0.003	−0.010	−1.867	0.062

Note. ^†^ Wald test on t(52239).

## Discussion

The findings indicate that in a Norwegian context both women and men reduce their drinking from pre-pregnancy into pregnancy. Reduction was apparent for all three measures of alcohol consumption. First-time fathers reduced their alcohol consumption more than experienced fathers, from initially higher levels. However, the gap between the fathers and their pregnant partner was still higher among first-time parents, as compared to experienced parents.

The reduction for fathers was particularly noteworthy, since it is not in line with previous studies with US samples, where most fathers were not found to reduce their alcohol consumption when their partner became pregnant [3,4,15,16]. Even though USA is mainly characterized by a “dry” alcohol culture, similar to Norway [40,41], there may still be cultural differences with relevance for expectant fathers’ alcohol consumption, notably the seemingly higher level of gender equality in Norway [18] as compared to in the USA [42]. Particularly of relevance is the extent to which men involve themselves in the care of their infants and small children, which characterizes an increasing number of Norwegian fathers [43], and which may very well also apply to involvement in various kinds of preparation for birth and care of their pregnant partner. Thus, Norwegian fathers’ strong tendency to involve themselves in childcare might explain their reduction in drinking in the transition to parenthood.

In order to encourage mothers to follow health instructions and abstain from alcohol during pregnancy, it has been argued that fathers ought to reduce their alcohol consumption as well [3,21,44]. However, our findings do not indicate any strong influence of fathers’ drinking on mothers’ drinking pattern, or the other way around. Although there was a weak to moderate association between reduced alcohol consumption of mothers and fathers, each parent’s change can be explained only to a small extent by the other parent’s drinking pattern. Thus, parents seem to make adaptations to pregnancy according to some individual standards and expectations, and these standards and expectations may account for the relatively weak relationship between the partners’ alcohol use. For a pregnant woman, responsibility for the fetus naturally puts strong pressure on her to reduce her alcohol intake, or preferably to abstain from alcohol and this pressure may outweigh any influence of her partner. For the father, there is no corresponding pressure. Still, fathers reduced their drinking to a considerable degree when their partner became pregnant, suggesting that they may have a gender-specific adaptation to the pregnancy. This corresponds well with findings from studies of mental health and well-being of men during this transitional period [45], as well as with classical role theory indicating reduced alcohol use along with a higher number of social roles [17]. Previous studies have shown that, particularly in countries with high gender equality, daily alcohol use decreases as female social roles increase [46]. The present findings may indicate a similar association among men.

No associations or only weak associations between drinking pre-pregnancy and relationship satisfaction in pregnancy were observed. The lack of any strong associations might be contingent of the context in which all couples experienced a pregnancy. In keeping with this, previous studies show that couples are generally quite satisfied with their relationship during pregnancy [47], suggesting that pregnancy may somehow protect both partners against relationship stress. Furthermore, associations between drinking and relationship processes have proved to be rather complex in some studies [48,49]. The notion of drinking partnership within married couples suggested by Roberts and Leonard [49] captures some of this complexity in which consumption levels and frequencies for both partners were taken into consideration, along with their tendencies to drink together or alone, at home or outside the home. More recently Levitt & Cooper [48] have used daily reports from young couples of quite similar aspects of drinking, in order to assess associations with relationship functions. Thus, stronger associations might have emerged if a more comprehensive measure of drinking partnership had been available in the present study.

More generally, a limitation of the present study is that drinking before pregnancy was assessed retrospectively. Questions assessing drinking in the past are generally thought to be less reliable than assessments of current drinking patterns because they are subject to recall bias. The further in the past that the behaviour occurred the stronger this effect will be [50]. In our study the past is only a few months ago, and thus it is not likely that any recall bias would influence the parents’ responses to a considerable degree. Moreover, self-reports of alcohol use seem to have acceptable reliability and validity in community populations [51], even when measured by only one or a few items [52] and this probably applies to our study as well. A study among pregnant women found no significant difference in self-reported alcohol consumption obtained by confidential or anonymous questionnaires [53], which may indicate that the social desirability of reporting low levels of alcohol use did not influence the pregnant women’s responses. It is not likely that this would be very different for the expectant fathers. However, it cannot be ruled out that the reported consumption level might be influenced by the participants’ perceived need of being in accordance with their inner values concerning alcohol use and pregnancy. However, any such effects may be more likely among mothers than fathers.

Alcohol use before pregnancy was assessed using gender-specific periods (men 6 months, women only 3 months). This may have influenced parents’ responses to this item differently. Mothers may have decreased their alcohol use already in expectation of becoming pregnant, suggesting even larger changes in their alcohol consumption than indicated in this study. It is also a shortcoming that data for estimating change were limited to two time points. Additional data collections could have strengthened the design. Another shortcoming was the lack of representation among the lowest and highest education groups. This poses a threat to the external validity of the findings. However, although the variability in education level was relatively large, education was only weakly related to drinking and relationship satisfaction. This may indicate that education has a limited influence on the present research issues. Other variables that were not measured might also differentiate between responders and those who refused to participate in the study.

Importantly, the analysis of participation of the fathers revealed selection effects related to transitional status, and mothers’ alcohol use, in that first-time fathers were more likely to participate than experienced fathers, and fathers with partners with lower alcohol consumption levels were more likely to participate. Such selection effects might bias estimates and limit the generalizability of findings. However, the probability of bias due to selective participation of first-time parents is not strong, since all analyses were stratified by transitional status. Building on a missing at random (MAR) mechanism for participation, the selection due to partners’ alcohol consumption was reduced by including partners’ alcohol consumption in all the models.

Acknowledging these potential limitations, the study still provides valuable information about drinking patterns during pregnancy, particularly the extent to which fathers reduce their alcohol consumption during this period of change. This has seldom been studied in large population groups. The substantial reduction in expectant fathers’ alcohol consumption was a robust finding and is not likely to be due to any methodological limitations of the study. The present study was carried out using a large community sample, whereas much of the research within this field has been carried out using relatively small and non-representative samples. Moreover, we were able to distinguish between couples in the transition to parenthood, and couples who merely experienced another pregnancy, which is rare in this research field. More studies are needed to replicate these findings in other cultural settings, and to provide more knowledge about why fathers seem to reduce their alcohol consumption when their partner becomes pregnant.

Our findings may have important policy implications, since they reveal a relatively strong reduction in alcohol consumption among all expectant fathers, and particularly first-time fathers. Although the individual benefits are probably limited, at a population level the establishment of more healthy alcohol patterns in the transition to parenthood might contribute to somewhat lower risks for the harmful effects of alcohol use, both for the fathers, the couple’s relationship, and the children, in terms of e.g. domestic violence, injuries and driving under the influence of alcohol. Thus the partner’s pregnancy may provide a golden opportunity for addressing men’s alcohol consumption.

## Conclusions

In a culture characterized by relatively high gender equality, men seem to reduce their alcohol use to a considerable degree when their partner becomes pregnant. This applies particularly to men in the transition to parenthood. The change in fathers’ alcohol use was only slightly related to reduced alcohol consumption of their pregnant partner. Thus fathers seem to make individual adaptations to their partners’ pregnancy, which may indicate that they develop a distinct social role as expectant father during the pregnancy. This might have important policy implications for alcohol preventive measures.

## Abbreviations

HED: Heavy episodic drinking; RS: Relationship satisfaction; AU: Alcohol units; APIM: Actor-partner-interdependence model; FIML: Full maximum likelihood; MAR: Missing at random; WLSMV: Weighted least squares means and variance.

## Competing interests

There are no competing interests that we are aware of.

## Authors’ contributions

SM has made a substantial contribution to the conception and design of the study, analysis and interpretation of the data and drafting and critical revision of the manuscript. TT has made a substantial contribution to the conception and design of the study, analysis and interpretation of data and drafting the manuscript. FT has made a substantial contribution to acquisition of the data and revising the paper. All authors have given final approval of the version to be published.
